# Albumin-Bilirubin Score: A Novel Mortality Predictor in Valvular Surgery

**DOI:** 10.21470/1678-9741-2022-0008

**Published:** 2023

**Authors:** Zihni Mert Duman, Barış Timur

**Affiliations:** 1 Department of Cardiovascular Surgery, Cizre State Hospital, Şırnak Turkey; 2 Department of Cardiovascular Surgery, Dr Siyami Ersek Thoracic and Cardiovascular Surgery Training and Research Hospital, İstanbul, Turkey

**Keywords:** Heart Valve, Acute Kidney Injury, Albumins, Aptitude, Echocardiography, Hospital Mortality, Preoperative Period.

## Abstract

**Introduction:**

The heart and liver are two organs that are closely related. The Albumin-Bilirubin (ALBI) score is a developed scoring system for assessing liver function. The aims of this study were to examine the correlation between preoperative ALBI score and pulmonary artery pressure and to investigate its ability to predict heart valve surgery mortality outcomes.

**Methods:**

The data of 872 patients who underwent isolated and combined heart valve surgery from 2014 to 2021 were retrospectively screened. In the preoperative period, 152 patients with laboratory tests including albumin and total bilirubin were found and analyzed retrospectively. Thirteen of these patients were excluded from the study. The remaining 139 patients were included in the analysis. Baseline demographic data, echocardiography data, performance status, laboratory data, operative data, and postoperative status were collected. The optimal cutoff value of preoperative ALBI score was calculated.

**Results:**

The cutoff for ALBI scores was calculated as -2.44 to predict in-hospital mortality (sensitivity = 75.0%, specificity = 70%). Based on the cutoff value, 90 patients had a low ALBI score (≤ -2.44, 64.7%) and 49 patients had a high ALBI score (> -2.44, 35.3%). High ALBI score was associated with an increased incidence of acute kidney injury and in-hospital mortality, and a positive correlation was found between ALBI score and pulmonary artery pressure.

**Conclusion:**

In patients with valvular surgery, high ALBI score was an independent prognostic factor of in-hospital mortality and acute kidney injury. It is easily measurable and a cost-effective way to predict mortality.

## INTRODUCTION

The heart and liver are two organs that are closely related. Liver failure may develop in patients with right heart failure due to pulmonary arterial hypertension^[^1^]^. The clinical and prognostic value of the interaction between the heart and liver is not clearly known, and some studies show that the Model for End-Stage Liver Disease (MELD) score, which is used for risk assessment in patients with liver failure, can also be used for risk assessment in cardiac surgery patients^[^2^-^4^]^.

The Albumin-Bilirubin (ALBI) score was recommended by Johnson et al.^[^5^]^ as an alternative to the Child-Pugh (or C-P) grade and the MELD score for risk assessment of liver function and subsequent long-term mortality in patients with liver disease. The ALBI score is a simpler test used to evaluate liver function that includes only serum albumin and bilirubin levels. This score is easier to calculate than the MELD score, and one of its advantages is that it is not affected by warfarin usage. In addition, the ALBI score is closely associated with hospital mortality in patients with heart failure as demonstrated in recent studies^[^6^,^7^]^. The ALBI score may be appropriate for preoperative risk analysis and evaluation of right heart failure due to pulmonary arterial hypertension in patients undergoing heart valve surgery.

The aims of this study were to examine the correlation between preoperative ALBI score and pulmonary artery pressure (PAP) and to investigate its ability to predict heart valve surgery mortality outcomes.

## METHODS

Patients who underwent only valvular surgery were included in the study. The data of 872 patients who had undergone isolated and combined heart valve surgery from 2014 to 2021 in Istanbul Mehmet Akif Ersoy Thoracic and Cardiovascular Surgery Training and Research Hospital were retrospectively screened. In the preoperative period, 152 patients with laboratory tests including albumin and total bilirubin were found and analyzed retrospectively. Exclusion criteria for this study were patients with known liver disease including positive hepatitis B antigen and anti-hepatitis C virus antigen and the patients who had to be operated in shock state or sepsis. Thirteen of these patients were excluded from the study, because they satisfied at least one of the exclusion criteria. The remaining 139 patients were included in the study to be analyzed. The baseline demographic data, echocardiographic data, performance status, laboratory data, operative data, and postoperative status were comprehensively collected and analyzed. PAP values were estimated from echocardiography.

The formula used to calculate the ALBI score is: (albumin × -0.085) + (log10 bilirubin × 0.66), where albumin is measured in g/L and bilirubin in µmol/L, as it was previously described in the literature^[^5^]^. The primary outcome was in-hospital mortality. Secondary outcomes included other postoperative complications, acute kidney injury (AKI), pneumonia, and re-exploration for bleeding. We used Standard Society of Thoracic Surgeons definitions in the study^[^8^]^. Hospital mortality was defined as mortality occurring within 30 days postoperatively or without discharge.

### Statistical Analysis

Statistical analyses were carried out using IBM Corp. Released 2015, IBM SPSS Statistics for Windows, version 23.0, Armonk, NY: IBM Corp. Descriptive statistics are reported as percentage for categoric variables and mean±standard deviation for continuous variables. Categorical variables were compared by a chi-squared analysis or Fisher’s exact test. Normal and abnormal continuous variables were compared by Student’s *t*-test and Mann-Whitney U test. Receiver operating characteristic (ROC) analysis was performed to find the appropriate cutoff value for the preoperative ALBI score. The ability of the ALBI score to predict hospital mortality was assessed using the area under the ROC curve (AUC). Univariate analysis of hospital mortality was performed using logistic regression model. Multivariate analysis was performed with variables that were statistically significant in univariate analysis. The correlation between the variables was analyzed using the Pearson’s or Spearman’s correlation coefficient. Linear regression model was used for the relationship between PAP and ALBI score. Statistical tests were two-sided, and *P*-values < 0.05 were considered statistically significant.

Our study was found ethically appropriate according to the decision of the Health Sciences University Mehmet Akif Ersoy Training and Research Hospital Clinical Research Ethics Committee (dated 04.06.2021; file number 2021/49).

## RESULTS

After randomization and exclusion of patients, the remaining 139 patients were included into the study. Most of the patients were women (56.1%) and mean age was 55.6±12.51 years. Three valves in 14 (10.1%) patients, two valves in 70 (50.4%) patients, and one valve in 55 (39.5%) patients were either replaced or repaired. Fifteen patients undergone cardiac reoperation (10.8%). Table 1 shows different operation types that were performed on these patients.

**Table 1:** Distribution of operation types.

Type of surgery	N (%)
Isolated aortic valve surgery	15 (10.8%)
Isolated mitral valve surgery	38 (27.3%)
Combined mitral and tricuspid valve surgery	50 (36%)
Combined aortic and mitral valve surgery	20 (14.4%)
Combined aortic, mitral, and tricuspid valve surgery	16 (11.5%)
Reoperation	15 (10.8%)

**Fig. 1:**
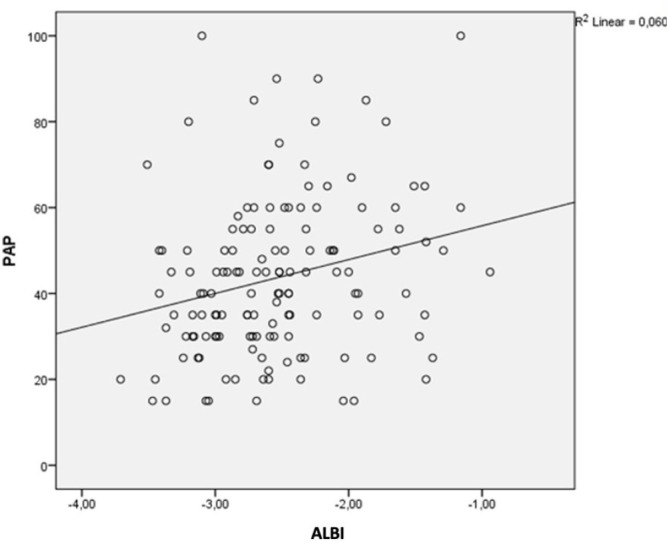
Correlation between the Albumin-Bilirubin (ALBI) score and the pulmonary artery pressure (PAP).

Mean ALBI score was -2.53±0.57 and median ALBI score was -2.59. Pearson’s correlation coefficients were used to analyze the relationship between ALBI score and PAP. We found a positive correlation (r: 0.245, *P*=0.004) between ALBI score and PAP. By linear regression analysis, we found that every 1 increase in ALBI score resulted in an increase in PAP of 7.95 mmHg (*P*=0.015). The graph is shown in Figure 1.

**Fig. 2:**
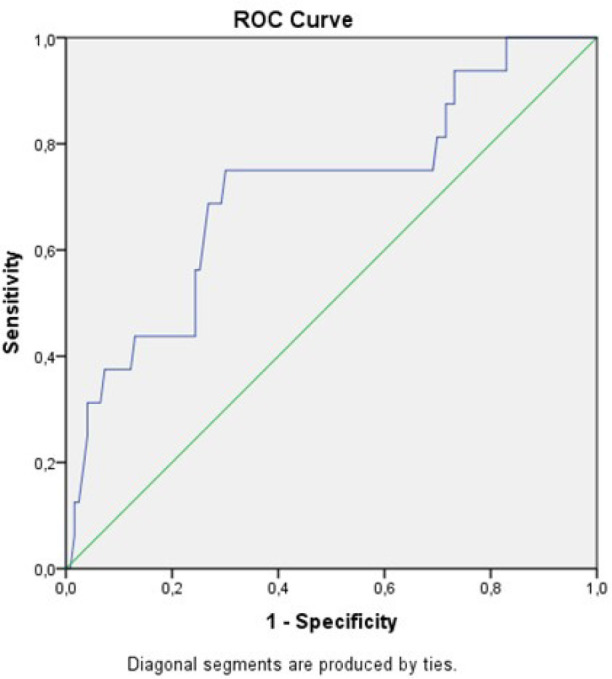
Receiver operating characteristic (ROC) analysis of optimal albumin-bilirubin value.

In-hospital mortality occurred in 16 (11.5%) patients. ROC curve analysis was established using the preoperative ALBI score to predict in-hospital mortality. The cutoff for ALBI scores was calculated as -2.44 for predicting in-hospital mortality (sensitivity = 75.0%, specificity = 70%, likelihood ratio: 2.5). The AUC was 0.712 and *P*-value was 0.004 for ROC curve analysis. Figure 2 shows that the preoperative ALBI score had a significant positive relationship with in-hospital mortality.

Seventeen of the 139 patients in the study were operated for infective endocarditis. Among patients with infective endocarditis, in-hospital mortality occurred in four patients. ROC curve analysis was done using the preoperative ALBI score to predict in-hospital mortality, excluding patients with infective endocarditis. The AUC was 0.697 and *P*-value was 0.025 for ROC curve analysis for this group of patients.

According to the cutoff value, 49 patients had a high ALBI score (ALBI > -2.44, 35.3%) and 90 patients had a low ALBI score (ALBI ≤ -2.44, 64.7%). Preoperative laboratory values and demographic data of patients with low and high ALBI scores are shown in Table 2. Patients with New York Heart Association (NYHA) class 3-4 symptoms were more common in the high ALBI score group (*P*<0.001). Patients with a high European System for Cardiac Operative Risk Evaluation (EuroSCORE) II were more common in the group with high ALBI scores (*P*<0.037). Also, patients who had valve surgery for infective endocarditis were more common in the high ALBI score group (*P*=0.007). Although there was no difference in ejection fraction between the two groups in echocardiographic data, PAPs were higher in the group with higher ALBI scores (*P*=0.011). Compared to the preoperative laboratory values, only the international normalized ratio values were higher in the high ALBI score group (*P*=0.048). The rest of the demographical and laboratory data were statistically insignificant (*P*>0.05).

**Table 2:** Preoperative demographic and clinical characteristics of the patients according to their ALBI scores.

	ALBI score < -2.44 (n=90) N (%) or mean±standard deviation	ALBI score >-2.44 (n=49) N (%) or mean±standard deviation	*P*-value
Age (years)	55.32±12.665	56.12±12.360	0.805
Sex (female)	54 (60%)	24 (48.9%)	0.211
NYHA class 3-4	36 (40%)	29 (59.1%)	0.00^*^
Preoperative atrial fibrillation	22 (24.4%)	18 (36.7%)	0.148
Infective endocarditis	6 (6.6%)	11 (22.4%)	0.007^*^
LVEF	54.77±9.44	55.20±9.62	0.727
PAP	40.91±17.31	48.86±19.66	0.011^*^
EuroSCORE II	2.40±1.88	4.22±5.11	0.037^*^
Reoperation	8 (9%)	7 (14%)	0.327
DM	22 (24.4%)	9 (18.3%)	0.411
Renal failure	13 (14.4%)	9 (18.3%)	0.545
COPD	22 (24.4%)	12 (24.5%)	0.995
Preoperative laboratory values			
WBC (109/L)	8.08±1.99	9.63±3.48	0.244
Hgb	19.51±11.42	17.10±11.28	0.313
PLT	236.88±63.87	279.08±57.55	0.090
ALT (U/L)	22.33±15.57	30.04±29.96	0.226
AST (U/L)	24.11±13.27	35.33±36.482	0.143
BUN	20.32±8.95	26.46±19.721	0.159
CRE	1.16±1.87	1.20±1.76	0.502
Hba1c	5.95±1.65	5.81±1.31	0.761
APTT (s)	29.88±6.46	30.16±5.71	0.301
INR	1.32±0.74	1.41±0.59	0.048^*^

^*^P-value < 0.05 is statistically significant

ALBI=Albumin-Bilirubin; ALT=alanine aminotransferase; APTT=activated partial thromboplastin time; AST=aspartate aminotransferase; BUN=blood urea nitrogen; COPD=chronic obstructive pulmonary disease; CRE=creatinine; DM=diabetes mellitus; EuroSCORE=European System for Cardiac Operative Risk Evaluation; Hba1c=glycosylated hemoglobin; Hgb=hemoglobin; INR=international normalized ratio; LVEF=left ventricular ejection fraction; NYHA=New York Heart Association; PAP=pulmonary artery pressure; PLT=platelet; WBC=white blood cell

Perioperative data of patients with low and high ALBI scores are shown in Table 3. There was no statistical difference between re-exploration for bleeding, cross-clamping time, and cardiopulmonary bypass (CPB) time (*P*>0.05).

**Table 3:** Comparison of the operative data of the patients according to the ALBI score greater and lesser than -2.44.

	ALBI score < -2.44 (n=90) N (%) or mean±standard deviation	ALBI score > -2.44 (n=49) N (%) or mean±standard deviation	*P*-value
CC time	90.59±36.180	96.04±36.487	0.371
CPB time	134.28±48.12	147.94±65.219	0.406
Re-exploration for bleeding	17 (18.9%)	5 (10.2%)	0.180

ALBI=Albumin-Bilirubin; CC=cross-clamping; CPB=cardiopulmonary bypass

Univariable and multivariable analyses were performed to identify independent risk factors related to in-hospital mortality. High preoperative ALBI score (odds ratio [OR]: 3.83, *P*=0.004), NYHA class 3-4 symptoms (OR: 4.51, *P*=0.013), high EuroSCORE II (OR: 1.25, *P*=0.002), CPB time (OR: 1.02, *P*<0.001), and combined aortic, mitral, and tricuspid valve surgery (OR: 6.78, *P*=0.002) were associated with hospital mortality in univariate analysis. Multivariate analysis using the stepwise model shows that high preoperative ALBI score (OR: 3.37, *P*=0.036) and CPB time (OR: 1.02, *P*=0.001) are independently associated with hospital mortality. Univariate and multivariate mortality analyses are detailed in Table 4.

**Table 4:** Univariable and multivariable analyses of mortality.

	Univariable analyses			Multivariable analyses		
	OR	95% CI	*P*-value	OR	95% CI	*P*-value
ALBI score	3.83	1.55 - 9.49	0.004^*^	3.37	1.09 - 10.5	0.036^*^
EuroSCORE II	1.25	1.09 - 1.44	0.002^*^	1.09	0.92 - 1.29	0.331
Age (years)	1.05	0.99 - 1.1	0.077			
NYHA class 3-4	4.51	1.38 - 14.86	0.013^*^	3.14	0.71 - 14.5	0.134
LVEF	0.98	0.93 - 1.04	0.542			
PAP	1.02	0.99 - 1.05	0.187			
DM	1.18	0.35 - 3.97	0.783			
Renal failure	2.83	0.87 - 9.17	0.082			
CPB time	1.02	1.01 - 1.03	< 0.001^*^	1.02	1.01 - 1.03	0.001^*^
Combined aortic, mitral, and tricuspid valve surgery	6.78	2.04 - 22.5	0.002^*^	1.23	0.31 - 6.99	0.851
Infective endocarditis surgery	2.82	0.79 - 10.1	0.109			
Reoperation	2.20	0.75 - 3.92	0.115			

^*^P-value < 0.05 is statistically significant

ALBI=Albumin-Bilirubin; CI=confidence interval; CPB=cardiopulmonary bypass; DM=diabetes mellitus; EuroSCORE=European System for Cardiac Operative Risk Evaluation; LVEF=left ventricular ejection fraction; NYHA=New York Heart Association; OR=odds ratio; PAP=pulmonary artery pressure

Higher ALBI score was associated with an increased incidence of AKI (*P*=0.028) and hospital mortality (*P*<0.001). However, no significant differences were found for other complications (*P*>0.05). Table 5 shows the results of postoperative complications according to ALBI score of -2.44.

**Table 5:** Comparison of postoperative complications according to the ALBI score greater and lesser than -2.44.

	ALBI score < -2.44 (n=90) N (%) or mean±standard deviation	ALBI score > -2.44 (n=49) N (%) or mean±standard deviation	*P*-value
Hospital mortality	4 (4.4%)	12 (24.5%)	< 0.001^*^
Ventilation time (days)	2.23±453	5.16±8.27	0.214
ICU stay (days)	3.89±7.93	7.31±11.46	0.234
Hospital stay (days)	11.83±9.75	15.14±16.38	0.667
Temporary pacemaker requirement	14 (15.6%)	7 (14.2%)	0.842
Permanent pacemaker requirement	8 (8.8%)	3 (6.1%)	0.564
AKI	19 (21.1%)	19 (38.8%)	0.028^*^
Wound complication	10 (11.1%)	6 (12.2%)	0.841
ECMO	0 (0%)	2 (4.1%)	0.054
IABP	3 (3.3%)	2 (4.1%)	0.821
Postoperative AF	16 (17.8%)	9 (18.4%)	0.931

^*^P-value < 0.05 is statistically significant.

AF=atrial fibrillation; AKI=acute kidney injury; ALBI=Albumin-Bilirubin; ECMO=extracorporeal membrane oxygenation; IABP=intra-aortic balloon pump; ICU=intensive care unit

## DISCUSSION

There are problems in liver functions due to chronic congestive hepatopathy in heart valve patients^[^9^]^. Abnormalities in liver function tests in the preoperative period affect the surgical condition of patients. Preoperative risk analysis and prediction of prognosis in patients are the cornerstones of surgical management. Problems in liver function tests are important risk factors that are not considered in the risk models used in cardiac surgery^[^10^]^. In our study, we used the ALBI score, a newly developed method for risk assessment of patients with liver disease, to evaluate the preoperative risk for mortality in heart valve surgery patients. The ALBI score, which uses only serum albumin and total bilirubin values, can be easily measured^[^5^]^. As demonstrated by other studies on the prognosis of liver diseases, the ALBI score is useful in determining the degree of liver dysfunction^[^11^,^12^]^.

In our study, we found that the optimal ALBI cutoff value was -2.44, analyzed by the ROC curve to hospital mortality. This value is very close to the cutoff value (-2.6) between grades 1 and 2 in hepatectomy patients. We found that patients who were operated for infective endocarditis had NYHA classes 3 and 4 complaints, and those who had high PAP had higher ALBI scores. Low albumin levels, a negative acute phase reactant, due to sepsis developing in infective endocarditis patients cause an increase in ALBI score^[^13^]^. As expected, in our study, ALBI scores were higher in infective endocarditis cases.

We found a positive correlation between ALBI score and PAP. Every 1 change in ALBI score causes a 7.95 mmHg change in PAP. The relationship between high PAP and mortality in valve surgery has been shown in studies^[^14^]^. The correlation between PAP and ALBI score shows that ALBI score can be used safely in valve surgery to predict mortality as well. Also, as it is known, ALBI score has been shown to reflect right heart failure and related liver dysfunction in patients with acute heart failure^[^15^]^. Therefore, we suggest the use of ALBI score preoperatively to assess surgical risk.

Our study is the first to demonstrate the use of the ALBI scoring system in heart valve surgery to our knowledge. In our study, it was observed that patients with high ALBI scores in multivariate analysis had a higher mortality rate. In multivariate analysis, the ALBI score is a stronger predictor of hospital mortality compared to EuroSCORE II. Also, we think that the ALBI score is clinically important in the preoperative risk assessment phase in heart valve surgery because since more than one third of our study population has a high ALBI score, it is a strong variable in predicting postoperative hospital mortality and is easily measurable.

ALBI is a scoring system that only includes albumin and bilirubin levels of a patient. It’s easy to measure and calculate. Therefore, it is valuable for cardiac valvular surgical patients to foresee and predict mortality. Both albumin and bilirubin values are reachable and easy measures to calculate ALBI score. Previous scoring systems such as MELD are poor predictors of outcomes for cardiac patients^[^2^]^. Therefore, ALBI is an easy and cost-effective way to predict the mortality and can be used confidently in valvular surgery patients.

### Limitations

Our study has some limitations. First, it only included data from a single-center and a small patient population. Due to the small population, hospital mortality and morbidity were relatively low. We did not investigate the long-term consequences of ALBI score. There is a need for multicenter and prospective studies to support the use of ALBI score, which may be a cost-effective way to predict mortality.

## CONCLUSION

ALBI is a strong variable in predicting hospital mortality and AKI in cardiac valvular surgery. It is easy to measure both albumin and bilirubin to calculate ALBI score. We recommend the use of ALBI score preoperatively to predict mortality.
