# ERRATUM

**DOI:** 10.21470/1678-9741-2022-0008e

**Published:** 2026-05-06

In the article “Albumin-Bilirubin Score: A Novel Mortality Predictor in Valvular Surgery”, DOI https://doi.org/10.21470/1678-9741-2022-0008, published in the Brazilian Journal of Cardiovascular Surgery, 38(2):271-277, the references section was incorrectly presented.

**In the published article, the references were**:

Hyde JA, Chinn JA, Graham TR. Platelets and cardiopulmonary bypass. Perfusion. 1998;13(6):389-407. doi:10.1177/026765919801300603.

Bønding Andreasen J, Hvas AM, Ravn HB. Marked changes in platelet count and function following pediatric congenital heart surgery. Paediatr Anaesth. 2014;24(4):386-92. doi:10.1111/pan.12347.

Ignjatovic V, Than J, Summerhayes R, Newall F, Horton S, Cochrane A, et al. The quantitative and qualitative responses of platelets in pediatric patients undergoing cardiopulmonary bypass surgery. Pediatr Cardiol. 2012;33(1):55-9. doi:10.1007/s00246-011-0079-5.

Kunitomo R, Tsurusaki S, Suzuki R, Takaji K, Moriyama S, Hagio K, et al. Predictive factors for platelet number after cardiopulmonary bypass and postoperative blood loss. ASAIO J. 2002;48(6):671-4. doi:10.1097/00002480-200211000-00018.

Kertai MD, Zhou S, Karhausen JA, Cooter M, Jooste E, Li YJ, et al. Platelet counts, acute kidney injury, and mortality after coronary artery bypass grafting surgery. Anesthesiology. 2016;124(2):339-52. doi:10.1097/ALN.0000000000000959.

Griffin BR, Bronsert M, Reece TB, Pal JD, Cleveland JC, Fullerton DA, et al. Thrombocytopenia after cardiopulmonary bypass is associated with increased morbidity and mortality. Ann Thorac Surg. 2020;110(1):50-7. doi:10.1016/j.athoracsur.2019.10.039.

Karhausen JA, Smeltz AM, Akushevich I, Cooter M, Podgoreanu MV, Stafford-Smith M, et al. Platelet counts and postoperative stroke after coronary artery bypass grafting surgery. Anesth Analg. 2017;125(4):1129-39. doi:10.1213/ANE.0000000000002187.

Dewar DC, Tarrant SM, King KL, Balogh ZJ. Changes in the epidemiology and prediction of multiple-organ failure after injury. J Trauma Acute Care Surg. 2013;74(3):774-9. doi:10.1097/TA.0b013e31827a6e69.

Gao S, Li Y, Diao X, Yan S, Liu G, Liu M, et al. Vacuum-assisted venous drainage in adult cardiac surgery: a propensity-matched study. Interact Cardiovasc Thorac Surg. 2020;30(2):236-42. doi:10.1093/icvts/ivz253.

Spiezia L, Di Gregorio G, Campello E, Maggiolo S, Bortolussi G, Stellin G, et al. Predictors of postoperative bleeding in children undergoing cardiopulmonary bypass: a preliminary Italian study. Thromb Res. 2017;153:85-9. doi:10.1016/j.thromres.2017.03.021.

Stravitz RT, Ellerbe C, Durkalski V, Reuben A, Lisman T, Lee WM, et al. Thrombocytopenia is associated with multi-organ system failure in patients with acute liver failure. Clin Gastroenterol Hepatol. 2016;14(4):613-20.e4. doi:10.1016/j.cgh.2015.09.029.

Nydam TL, Kashuk JL, Moore EE, Johnson JL, Burlew CC, Biffl WL, et al. Refractory postinjury thrombocytopenia is associated with multiple organ failure and adverse outcomes. J Trauma. 2011;70(2):401-6. doi:10.1097/TA.0b013e31820b5c85.

Greco E, Lupia E, Bosco O, Vizio B, Montrucchio G. Platelets and multi-organ failure in sepsis. Int J Mol Sci. 2017;18(10):2200. doi:10.3390/ijms18102200.

Jansen MPB, Florquin S, Roelofs JJTH. The role of platelets in acute kidney injury. Nat Rev Nephrol. 2018;14(7):457-71. doi:10.1038/s41581-018-0015-5.

Bdeir K, Gollomp K, Stasiak M, Mei J, Papiewska-Pajak I, Zhao G, et al. Platelet-specific chemokines contribute to the pathogenesis of acute lung injury. Am J Respir Cell Mol Biol. 2017;56(2):261-70. doi:10.1165/rcmb.2015-0245OC.

Spiess BD. Platelets reflect biologic complexity. Anesthesiology. 2016;124(2):265-6. doi:10.1097/ALN.0000000000000960.

Karkouti K, Wijeysundera DN, Yau TM, Beattie WS, Abdelnaem E, McCluskey SA, et al. The independent association of massive blood loss with mortality in cardiac surgery. Transfusion. 2004;44(10):1453-62. doi:10.1111/j.1537-2995.2004.04144.x.

Karkouti K. Transfusion and risk of acute kidney injury in cardiac surgery. Br J Anaesth. 2012;109 Suppl 1:i29-i38. doi:10.1093/bja/ aes422.

Leytin V, Allen DJ, Mykhaylov S, Mis L, Lyubimov EV, Garvey B, et al. Pathologic high shear stress induces apoptosis events in human platelets. Biochem Biophys Res Commun. 2004;320(2):303-10. doi:10.1016/j.bbrc.2004.05.166.

Tran PL, Pietropaolo MG, Valerio L, Brengle W, Wong RK, Kazui T, et al. Hemolysate-mediated platelet aggregation: an additional risk mechanism contributing to thrombosis of continuous flow ventricular assist devices. Perfusion. 2016;31(5):401-8. doi:10.1177/0267659115615206.

Mohebali D, Kaplan D, Carlisle M, Supiano MA, Rondina MT. Alterations in platelet function during aging: clinical correlations with thromboinflammatory disease in older adults. J Am Geriatr Soc. 2014;62(3):529-35. doi:10.1111/jgs.12700.

Balduini CL, Noris P. Platelet count and aging. Haematologica. 2014;99(6):953-5. doi:10.3324/haematol.2014.106260.

Leguyader A, Watanabe R, Berbé J, Boumediene A, Cogné M, Laskar M. Platelet activation after aortic prosthetic valve surgery. Interact Cardiovasc Thorac Surg. 2006;5(1):60-4. doi:10.1510/ icvts.2005.115733.

Mahmood S, Bilal H, Zaman M, Tang A. Is a fully heparin-bonded cardiopulmonary bypass circuit superior to a standard cardiopulmonary bypass circuit? Interact Cardiovasc Thorac Surg. 2012;14(4):406-14. doi:10.1093/icvts/ivr124.

Jacobson J. Nitric oxide: platelet protectant properties during cardiopulmonary bypass/ECMO. J Extra Corpor Technol. 2002;34(2):144-7.

Selleng S, Selleng K. Heparin-induced thrombocytopenia in cardiac surgery and critically ill patients. Thromb Haemost. 2016;116(5):843-51. doi:10.1160/TH16-03-0230.

**The correct references are**:

Møller S, Bernardi M. Interactions of the heart and the liver. Eur Heart J. 2013;34(36):2804-11. doi:10.1093/eurheartj/eht246.

Assenza GE, Graham DA, Landzberg MJ, Valente AM, Singh MN, Bashir A, et al. MELD-XI score and cardiac mortality or transplantation in patients after fontan surgery. Heart. 2013;99(7):491-6. doi:10.1136/heartjnl-2012-303347.

Ailawadi G, Lapar DJ, Swenson BR, Siefert SA, Lau C, Kern JA, et al. Model for end-stage liver disease predicts mortality for tricuspid valve surgery. Ann Thorac Surg. 2009;87(5):1460-7; discussion 1467-8. doi:10.1016/j.athoracsur.2009.01.043.

Matthews JC, Pagani FD, Haft JW, Koelling TM, Naftel DC, Aaronson KD. Model for end-stage liver disease score predicts left ventricular assist device operative transfusion requirements, morbidity, and mortality. Circulation. 2010;121(2):214-20. doi:10.1161/CIRCULATIONAHA.108.838656.

Johnson PJ, Berhane S, Kagebayashi C, Satomura S, Teng M, Reeves HL, et al. Assessment of liver function in patients with hepatocellular carcinoma: a new evidence-based approach-the ALBI grade. J Clin Oncol. 2015;33(6):550-8. doi:10.1200/JCO.2014.57.9151.

Han S, Wang C, Tong F, Li Y, Li Z, Sun Z, et al. Prognostic impact of albumin-bilirubin score on the prediction of in-hospital mortality in patients with heart failure: a retrospective cohort study. BMJ Open. 2022;12(1):e049325. doi:10.1136/bmjopen-2021-049325.

Yamada S, Kaneshiro T, Yoshihisa A, Nodera M, Amami K, Nehashi T, et al. Albumin-bilirubin score for prediction of outcomes in heart failure patients treated with cardiac resynchronization therapy. J Clin Med. 2021;10(22):5378. doi:10.3390/jcm10225378.

The Society of Thoracic Surgeons. STS Adult Cardiac Surgery Database Data Specifications. Version 4.20.2. Chicago (IL): The Society of Thoracic Surgeons; c2022. Available at: https://www.sts.org/sites/default/files/ACSD_DataSpecifications_V4_20_2.pdf

Laribi S, Mebazaa A. Cardiohepatic syndrome: liver injury in decompensated heart failure. Curr Heart Fail Rep. 2014;11(3):236-40. doi:10.1007/s11897-014-0206-8.

Hawkins RB, Young BAC, Mehaffey JH, Speir AM, Quader MA, Rich JB, et al. Model for end-stage liver disease score independently predicts mortality in cardiac surgery. Ann Thorac Surg. 2019;107(6):1713-9. doi:10.1016/j.athoracsur.2018.12.011.

Ma T, Li QS, Wang Y, Wang B, Wu Z, Lv Y, et al. Value of pretransplant albumin-bilirubin score in predicting outcomes after liver transplantation. World J Gastroenterol. 2019;25(15):1879-89. doi:10.3748/wjg.v25.i15.1879.

Zhang ZQ, Xiong L, Zhou JJ, Miao XY, Li QL, Wen Y, et al. Ability of the ALBI grade to predict posthepatectomy liver failure and long-term survival after liver resection for different BCLC stages of HCC. World J Surg Oncol. 2018;16(1):208. doi:10.1186/s12957-018-1500-9.

Arques S. Human serum albumin in cardiovascular diseases. Eur J Intern Med. 2018;52:8-12. doi:10.1016/j.ejim.2018.04.014.

Schubert SA, Mehaffey JH, Booth A, Yarboro LT, Kern JA, Kennedy JLW, et al. Pulmonary-systemic pressure ratio correlates with morbidity in cardiac valve surgery. J Cardiothorac Vasc Anesth. 2019;33(3):677-82. doi:10.1053/j.jvca.2018.08.190.

Matsue Y, Kagiyama N, Yamaguchi T, Kuroda S, Okumura T, Kida K, et al. Clinical and prognostic values of ALBI score in patients with acute heart failure. Heart Lung Circ. 2020;29(9):1328-37. doi:10.1016/j.hlc.2019.12.003.

The Brazilian Journal of Cardiovascular Surgery apologizes for this error, which has now been corrected.

